# The Clinics of John B. Murphy

**Published:** 1916-03

**Authors:** 


**The Clinics of John B. Murphy** at the Mercy Hospital, Chicago, December, 1915.

In the discussion following two operations for undescended testicle, Dr. Murphy refers to the paper of Armstrong of Wellington College (in Med. Press and Circ., Aug. 4, 1915), in which he reports remarkable results from the administration of thyroid extract in cases of undescended testicle, and quotes him as follows: "During the last four years out of 600 boys of the age of 13 and 14, 4 were found with complete absence of both testicles from the scrotum. In each case the penis was very small and there was no appearance of hair on the pubis. Two of them had Levi de Rothschild's sign of thyroid inadequacy—i.e. rarefaction of the outer third of the eyebrows. One was of the Mongolian type.

"To each of them thyroid extract in ½ gr. doses was given twice daily over a considerable period, with satisfactory results. In a few weeks the testicles could be felt in the inguinal canal, and in 3 of the 4 cases the complete descent into the scrotum was established in about three months. In the fourth case one testicle is now completely and the other almost descended."

Until this is established, all cases of undescended testicle are to be operated upon except, says Dr. Murphy, in those cases in which the testicle is situated within the abdomen. The organ may be fixed in the scrotum, removed from the body or returned to the abdomen.

The chance of an undescended testicle becoming sarcomatous is very great. Even in those not subject to trauma, as for instance those within the abdominal cavity. In fact, if one testicle is removed for sarcamatous change the remaining one has frequently been known to be sarcomatous also. Similar observations are made in other paired organs, as kidneys and ovary. Malignancy may be explained by Cohnheim's theory, or better perhaps by being due to the influence of some biochemical agent.

This number deals with other congenital deformities as cervical ribs, thyroglossal duct, congenital nasal deformity, etc., and a large variety of other lesions besides the usual bone and joint cases.—C. W. H.

